# Supplementary material for: Fish larval recruitment to reefs is a thyroid hormone-mediated metamorphosis sensitive to the pesticide chlorpyrifos
Source: eLife. 2017 Oct 30;6:e27595. doi: 10.7554/eLife.27595 (PMC5662287; doi:10.7554/eLife.27595)
Supplement: Transparent reporting form [file elife-27595-transrepform.pdf]

eLIFE
1st Floor
24 Hills Road
Cambridge CB2 1JP, UK
P 01223 855340
W elifesciences.org
T @elife

eLife's transparent reporting form

We encourage authors to provide detailed information *within their submission* to facilitate the interpretation and replication of experiments. If you have any questions, please contact us:editorial@elifesciences.org.

Sample-size estimation

- You should state whether an appropriate sample size was computed when the study was being designed
- You should state the statistical method of sample size computation and any required assumptions
- If no explicit power analysis was used, you should describe how you decided what sample (replicate) size (number) to use

Please outline where this information can be found within the submission (e.g., page numbers or figure legends), or explain why this information doesn't apply to your submission:

We indicated the sample size in our text and in our figure legends:
- The sample size are indicated in the legend for all the panels of the figure 1, page 17 lines 609, 610 and 617 to 623.
- p3, line 81, within the results text, for the figure 1B.
- The sample size are indicated in the legend for all the panels of the figure 2, page 20, line 643.
- p4, lines 124-125, within the results text, for the results of Figure 2A-B
- The sample size are indicated in the legend for all the panels of the figure 3, page 24, 679, 690, 691 and 694.
- The sample size are indicated in the legend for all the panels of the figure 4, page 28, line 715.
- The sample size are indicated in the legend for all the panels of the figure 5, page 29, line 725, 729 and 732.
- The sample size are indicated in the material end method for the figure 5, chlorpyrifos exporsure, page 10, line 346
- The sample size are indicated in the legend of the figure 1 – figure supplement 1, page 19, line 631, 632 and 639.
- The sample size are indicated in the legend of the figure 3 – figure supplement 1, page 26, line 702, 705 and 706.

The sample size are indicated in the legend of the figure 5 – figure supplement 2, page 30, line 742 and 744
Sample sizes were restricted to the availability of wild fish since we captured them *in situ*.

Replicates

- You should report how often each experiment was performed

- You should include a definition of biological versus technical replication
- The data obtained should be provided and sufficient information should be provided to indicate the number of independent biological and/or technical replicates
- If you encountered any outliers, you should describe how these were handled
- Criteria for exclusion/inclusion of data should be clearly stated
- High-throughput sequence data should be uploaded before submission, with a private link for reviewers provided (these are available from both GEO and ArrayExpress)

Please outline where this information can be found within the submission (e.g., page numbers or figure legends), or explain why this information doesn't apply to your submission:

Biological replicates are indicated as sample size (for more details on sample-size, see the previous paragraph). Technical replicate correspond to the number of time the experiment was performed from the same biological sample. No outlier was excluded in our analysis.

When technical replicates were performed (histology, qPCR and transactivation assays → laboratory analyses), we indicated it in our text:
- In the legend of the figure 1, page 18, line 614, 616 and 619.
- In the legend of the figure 2, page 20, line 648.
- In the legend of the figure 3, page 25, line 684, 691, 692 and 694.
- In the legend of the figure 1 – figure supplement 1, page 19, line 631 and 632.
- In the legend of the figure 2 – figure supplement 1, page 21, line 659, 662.
- In the legend of the figure 3 – figure supplement 1, page 26, line 705-706.
- In the legend of the figure 3 – figure supplement 2, page 27, line 709.
- In the legend of the figure 5 – figure supplement 2, page 30, line 744.

Statistical reporting

- Statistical analysis methods should be described and justified
- Raw data should be presented in figures whenever informative to do so (typically when N per group is less than 10)
- For each experiment, you should identify the statistical tests used, exact values of N, definitions of center, methods of multiple test correction, and dispersion and precision measures (e.g., mean, median, SD, SEM, confidence intervals; and, for the major substantive results, a measure of effect size (e.g., Pearson's r, Cohen's d)
- Report exact p-values wherever possible alongside the summary statistics and 95% confidence intervals. These should be reported for all key questions and not only when the p-value is less than 0.05.

Please outline where this information can be found within the submission (e.g., page numbers or figure legends), or explain why this information doesn't apply to your submission:

Statistical methods used for each result are indicated in the legend for each figure.
Errors bars indicate standard errors in each figure.
Statistical tests were performed with R.
We used Wilcoxon-Mann-Withney U-test when comparing two means, especially when n < 10.
For multiple mean comparisons, we assessed the values (or residuals) normality as well as the homogeneity of variances, and performed ANOVA analyses followed (if significant) by Tukey posthoc tests.
For qPCRs, a student's t cumulative distribution function was automatically computed by qPCR software CFX Biorad Manager.
We provided the exact p-values in our Results text, and present them using asterisks in our figures : *: p-value < 0.05; **: p-value < 0.01; ***: p-value < 0.001.

Detailed statistics (providing shapiro, Bartlett, Wilcoxon, anova and tukey precise and complete outcomes) are available in the source-data that we provided for all our graphs and all our results.

Within the submission, statistical tests that were used can be found:
- p17, line 610, within figure 1 legend, for figure 1B
- p18, line 621, within figure 1 legend, for figure 1W
- p20, line 644, within figure 2 legend, for figure 2A-B
- p20, line 649, within figure 2 legend, for figure 2C.
- p24, line 681, within figure 3 legend, for figure 3B-C
- p25, line 685, within figure 3 legend, for figure 3D
- p25, line 688, within figure 3 legend, for figure 3E
- p25, line 696, within figure 3 legend, for figure 3F-G
- p28, line 718, within figure 4 legend, for figure 4
- p29, line 726, within figure 5 legend, for figure 5A
- p29, line 729, within figure 5 legend, for figure 5B
- p29, line 733, within figure 5 legend, for figure 5C
- p29, line 737, within figure 5 legend, for figure 5D
- p12, lines 441-448, within Mat&Met text, for a description of each statistical test that was used within this manuscript.

Within the submission, p-values can be found:
- lines 127, 132, 144, 171, 173, 174, 176, 199, 200, 201, 210, 211, 227, 228, 231, 232, 234, 236, and 238.

(For large datasets, or papers with a very large number of statistical tests, you may upload a single table file with tests, Ns, etc., with reference to page numbers in the manuscript.)

eLife Sciences Publications, Ltd is a limited liability non-profit non-stock corporation incorporated in the State of Delaware, USA, with company number 5030732, and is registered in the UK with company number FC030576 and branch number BR015634 at the address 1st Floor, 24 Hills Road, Cambridge CB2 1JP | August 2014

eLIFE
1st Floor
24 Hills Road
Cambridge CB2 1JP, UK
P 01223 855340
W elifesciences.org
T @elife

Additional data files ("source data")

- We encourage you to upload relevant additional data files, such as numerical data that are represented as a graph in a figure, or as a summary table
- Where provided, these should be in the most useful format, and they can be uploaded as "Source data" files linked to a main figure or table
- Include model definition files including the full list of parameters used
- Include code used for data analysis (e.g., R, MatLab)
- Avoid stating that data files are "available upon request"

Please indicate the figures or tables for which source data files have been provided:

Source data have been provided for the figures:
Figure 1B (Figure 1 - data source 1)
Figure 1W (Figure 1 - data source 2)
Figure 2A (Figure 2 - data source 1)
Figure 2B (Figure 2 - data source 2)
Figure 2C (Figure 2 - data source 3)
Figure 2 - figure supplement 1C (Figure 2 - data source 4)
Figure 2 - figure supplement 1D (Figure 2 - data source 5)
Figure 2 – figure supplement 2 (Figure 2 - data source 6)
Figure 2 – figure supplement 3 (Figure 2 - data source 7)
Figure 3B (Figure 3 - data source 1)
Figure 3C (Figure 3 - data source 2)
Figure 3D (Figure 3 - data source 3)
Figure 3E (Figure 3 - data source 4)
Figure 3G (Figure 3 - data source 5)
Figure 4 (Figure 4 - data source 1)
Figure 5A (Figure 5 - data source 1)
Figure 5B (Figure 5 – data source 2)
Figure 5C (Figure 5 – data source 3)
Figure 5D (Figure 5 – data source 4)
Figure 5 - figure supplement 1A. (Figure 5 – data source 5)

eLife Sciences Publications, Ltd is a limited liability non-profit non-stock corporation incorporated in the State of Delaware, USA, with company number 5030732, and is registered in the UK with company number FC030576 and branch number BR015634 at the address 1st Floor, 24 Hills Road, Cambridge CB2 1JP | August 2014
